# Supplementary material for: Association between oral microbiome and seven types of cancers in East Asian population: a two-sample Mendelian randomization analysis
Source: Front Mol Biosci. 2023 Nov 21;10:1327893. doi: 10.3389/fmolb.2023.1327893 (PMC10702768; doi:10.3389/fmolb.2023.1327893)
Supplement: Supplementary file 1 [file DataSheet1.ZIP › Supplementary Materials/MR plots for tongue/tongue═╝/Prostate cancer/pheno.1430_to_prostate cancer_forest.pdf]

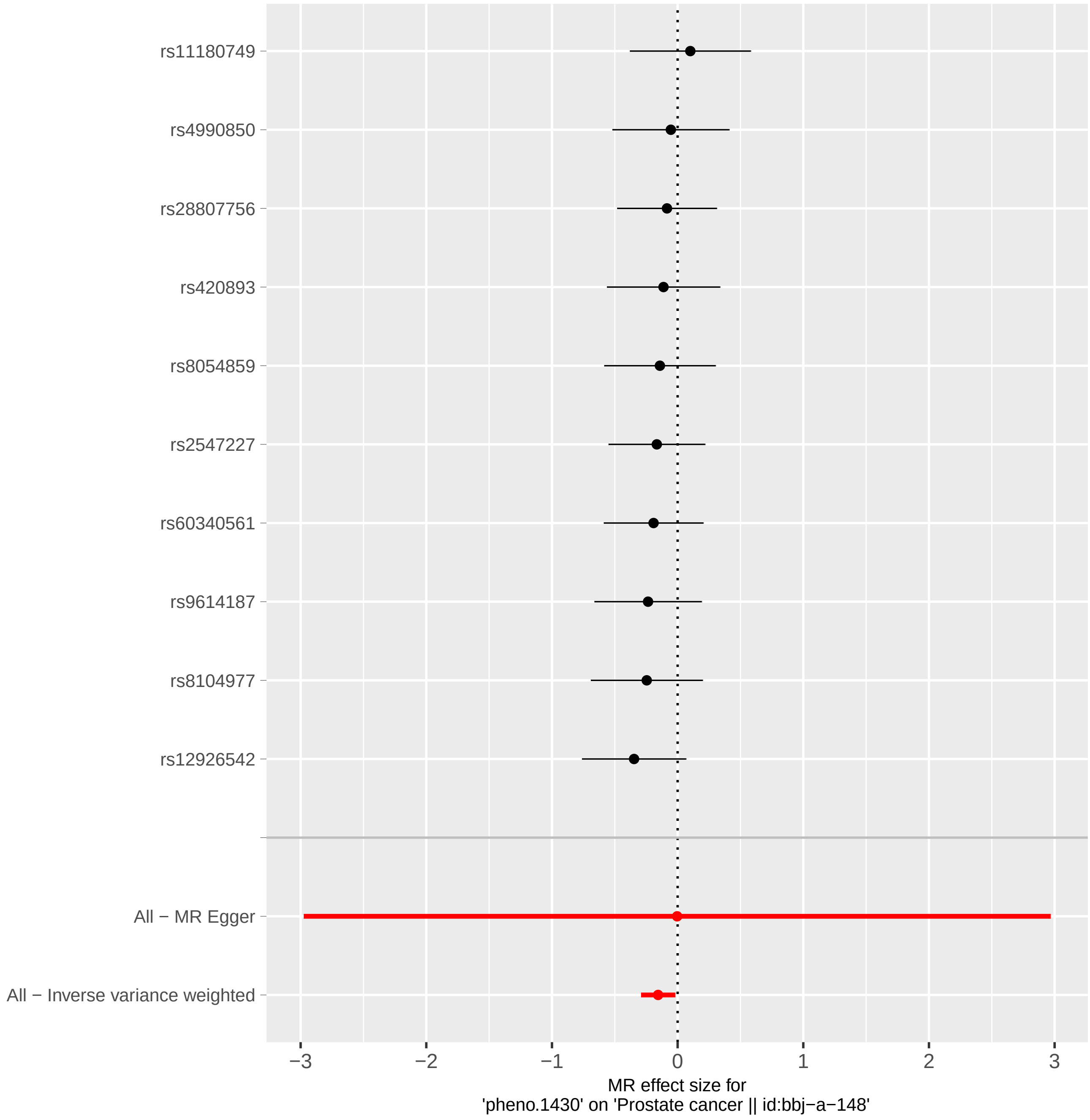
rs11180749
rs4990850
rs28807756
rs420893
rs8054859
rs2547227
rs60340561
rs9614187
rs8104977
rs12926542
All − MR Egger
All − Inverse variance weighted
−3
−2
−1
0
1
2
3
MR effect size for
'pheno.1430' on 'Prostate cancer || id:bbj−a−148'
